# Prevalence and predictors of post-stroke cognitive impairment among stroke survivors in Uganda

**DOI:** 10.1186/s12883-023-03212-8

**Published:** 2023-04-25

**Authors:** Martin N. Kaddumukasa, Mark Kaddumukasa, Elly Katabira, Nelson Sewankambo, Lillian D. Namujju, Larry B. Goldstein

**Affiliations:** 1 Department of Medicine, School of Medicine, College of Health Sciences, Makerere University, Kampala, Uganda; 2 Department of Electrical and Computer Engineering, College of Engineering, Design, Art and Technology, Makerere University, Kampala, Uganda; 3 Department of Neurology, University of Kentucky, Lexington, KY USA

**Keywords:** Stroke, Cognitive impairment, Dementia, Montreal Cognitive Assessment, Sub-Saharan Africa

## Abstract

**Background:**

Little is known about the characteristics and determinants of post-stroke cognitive impairment in residents of low- and middle-income countries. The objective of this study was to determine the frequencies, patterns, and risk factors for cognitive impairment in a cross-sectional study of consecutive stroke patients cared for at Uganda’s Mulago Hospital, located in sub-Saharan Africa.

**Methods:**

131 patients were enrolled a minimum of 3-months after hospital admission for stroke. A questionnaire, clinical examination findings, and laboratory test results were used to collect demographic information and data on vascular risk factors and clinical characteristics. Independent predictor variables associated with cognitive impairment were ascertained. Stroke impairments, disability, and handicap were assessed using the National Institute of Health Stroke Scale (NIHSS), Barthel Index (BI), and modified Rankin scale (mRS), respectively. The Montreal Cognitive Assessment (MoCA) was used to assess participants’ cognitive function. Stepwise multiple logistic regression was used to identify variables independently associated with cognitive impairment.

**Results:**

The overall mean MoCA score was 11.7-points (range 0.0–28.0-points) for 128 patients with available data of whom 66.4% were categorized as cognitively impaired (MoCA < 19-points). Increasing age (OR 1.04, 95% CI 1.00-1.07; p = 0.026), low level of education (OR 3.23, 95% CI 1.25–8.33; p = 0.016), functional handicap (mRS 3–5; OR 1.84, 95% CI 1.28–2.63; p < 0.001) and high LDL cholesterol (OR 2.74, 95% CI 1.14–6.56; p = 0.024) were independently associated with cognitive impairment.

**Conclusions:**

Our findings highlight the high burden and need for awareness of cognitive impairment in post stroke populations in the sub-Saharan region and serve to emphasize the importance of detailed cognitive assessment as part of routine clinical evaluation of patients who have had a stroke.

## Introduction

Stroke is a major cause of death and disability among adults worldwide, but particularly in low- and middle-income countries in sub-Saharan Africa [1–3]. Stroke is also the second most common cause of cognitive impairment with stroke survivors often having profound cognitive deficits [3]. Up to 64% of stroke survivors develop some degree of cognitive impairment and about 30% die with complications from dementia [4, 5]. Post-stroke cognitive impairment characteristically involves multiple domains including attention and concentration, executive function, language, memory, and visuospatial function, with executive function being affected the greatest [4, 6, 7]. Persons with mild cognitive impairment (MCI) convert to Alzheimer’s disease (AD) at an annual rate of 10–12% in contrast to 1–2% in the elderly population without MCI [8]. Cognitive impairment can have an important impact on quality of life and activities of daily living by reducing independence [6] and is associated with long-term morbidity and disability [9].

Although the prevalence of post-stroke cognitive impairment has been studied in different countries [10, 11], data are inconsistent due to differences in patient characteristics, neuro-psychological assessments, sample sizes, and analytical methods [12]. Accurate estimation of the prevalence of post-stroke cognitive impairment is limited by these and other factors with frequencies varying from 30 to 50% [13]. A myriad of risk factors have been proposed as predisposing to post-stroke vascular cognitive impairment including socio-demographic characteristics such as age, sex, educational attainment, occupation and environmental enrichment, cardiovascular risk factors, and stroke-related characteristics such as the extent and sites of brain injury [3, 4, 14, 15]. A meta-analysis of 65 observational studies, published in 2019, reported only limited data on cognitive impairment in Sub-Saharan Africa, but found overall prevalence of 30–40% in those age 5- years or older [16]. There are only limited data characterizing the burden, spectrum, determinants and consequences of post-stroke cognitive impairment in patients residing in low- and middle-income countries in sub-Saharan Africa [3]. In the present study, we assessed the frequency and risk factors associated with cognitive impairment 3-months after stroke in patients who received care in the primary referral hospital in Uganda.

## Methods

### Study design, setting and population

This was a cross-sectional study conducted at Mulago Hospital, Uganda’s main national referral hospital. Consecutive patients who had a first-ever or recurrent stroke and were admitted to the hospital between August 2019 - July 2020 were recruited. Stroke was defined as a neurologic deficit of abrupt onset attributable to a vascular cause with neurologic deficits lasting more than 24-hours and with compatible findings on CT brain scan [17, 18]. We classified ischemic stroke subtypes according to the Trial of Org 10,172 in Acute Stroke Treatment (TOAST) criteria [19]. Cognitive impairment was defined as a decline in function in either one or several domains of cognitive function as assessed by the Montreal Cognitive Assessment (MoCA) [20] evidenced by a MoCA score < 19.

Study participants were age > 18-years and had a minor-to-severe stroke (National Institutes of Health Stroke Scale [NIHSS] score between 1 and 25) and enrolled a minimum of 3-months after hospital admission for a stroke. Those whose stroke status could not be confirmed on CT scan and those with a history of severe cognitive impairment (dementia) prior to the index stroke, or a history of psychiatric disease (schizophrenia, manic-depressive disorder, and major depression) were excluded [13, 21].

### Data collection

A questionnaire was administered to collect demographic information including age, highest level of education, occupation, sex, work status, marital status and living conditions. Vascular risk factors were assessed based on self-report, reported use of key medications, and review of medical records. Cardiac disease including myocardial infarction and atrial fibrillation were assessed based on self-reported history, clinical examination, and review of both baseline and historical ECG results.

### Clinical assessments

All participants had a general physical examination after enrollment. Blood pressure was measured with an automated sphygmomanometer twice after an interval of 15-minutes with the subject seated and the average recorded. Hypertension was defined as BP > 140/90mmHg and/or receiving antihypertensive medications over a period longer than 1-month. Diabetes mellitus was diagnosed based on having a fasting blood glucose level > 126 mg/dl and/or receiving related medications. Dyslipidemia was defined as total cholesterol concentration > = 4.5mmol/L, and/or LDL cholesterol > = 2.5mmol/L [22], and/or use of a statin for dyslipidemia.

Stroke impairment and post-stroke handicap were assessed at enrollment using the NIHSS [23] and modified Rankin scale (mRS), respectively [24, 25] by trained research assistants based on retrospective medical record review and interview of stroke survivors and/or their proxy. NIHSS scores ranging from 1 to 4, 5–15 and 16–25 were defined as mild, moderate, and severe stroke respectively; mRS scores 0–2 and 3–5 corresponded to mild and severe post-stroke handicaps, respectively.

Assessment of patients’ dependency was based on the Barthel Index (BI) obtained on the day of enrollment and classified into three grades: Complete dependence (BI score < 60), moderate dependence or assisted independence (BI score 60–94), and minimal or no disability (BI score ≥ 95)[26].

The Montreal Cognitive Assessment (MoCA) [27, 28] was used to assess participants’ cognitive function. The MoCA includes eight cognitive domains (attention, concentration, memory, language, orientation, visuo-constructional skills, conceptual thinking, and calculation). Responses were scored according to specified criteria, with a maximum score of 30 points [13, 29]. Scores < 19 and > = 19 were categorized as reflecting cognitive impairment and no impairment, respectively. An optimal cutoff of 19 for the detection of mild cognitive impairment among minority Blacks with low level of education of < 12 years has been proposed [30]. To ensure the validity and reliability of the MoCA test, it was administered after rigorous training sessions with Luganda (local language)-speaking examiners, conducted with discussions with an expert panel of 2 neurologists, 1 psychologist and 1 educator. The phonemic fluency task and sentence repetition task were deemed problematic; thus were substituted a semantic fluency task (animal naming) for phonemic fluency, a strategy also used in the Korean version of the MoCA [20] .

### Statistical analysis

Descriptive statistics including means, frequencies and percentages were used to summarize participants’ cognitive data over different socio-demographic variable groups including age, sex, education level, living conditions, and work status. Univariable associations between the outcome variable, cognitive impairment, and categorical baseline variables were determined with chi-square tests; t-tests were used for continuous variables. Participant records with key missing data were excluded from the analysis. The results of the univariable analyses were subsequently adjusted for known confounders including age, sex and stroke severity [13] in a stepwise multiple logistic regression model using both forward and backward elimination to determine independent relationships with cognitive impairment.

## Results

**Fig. 1:**
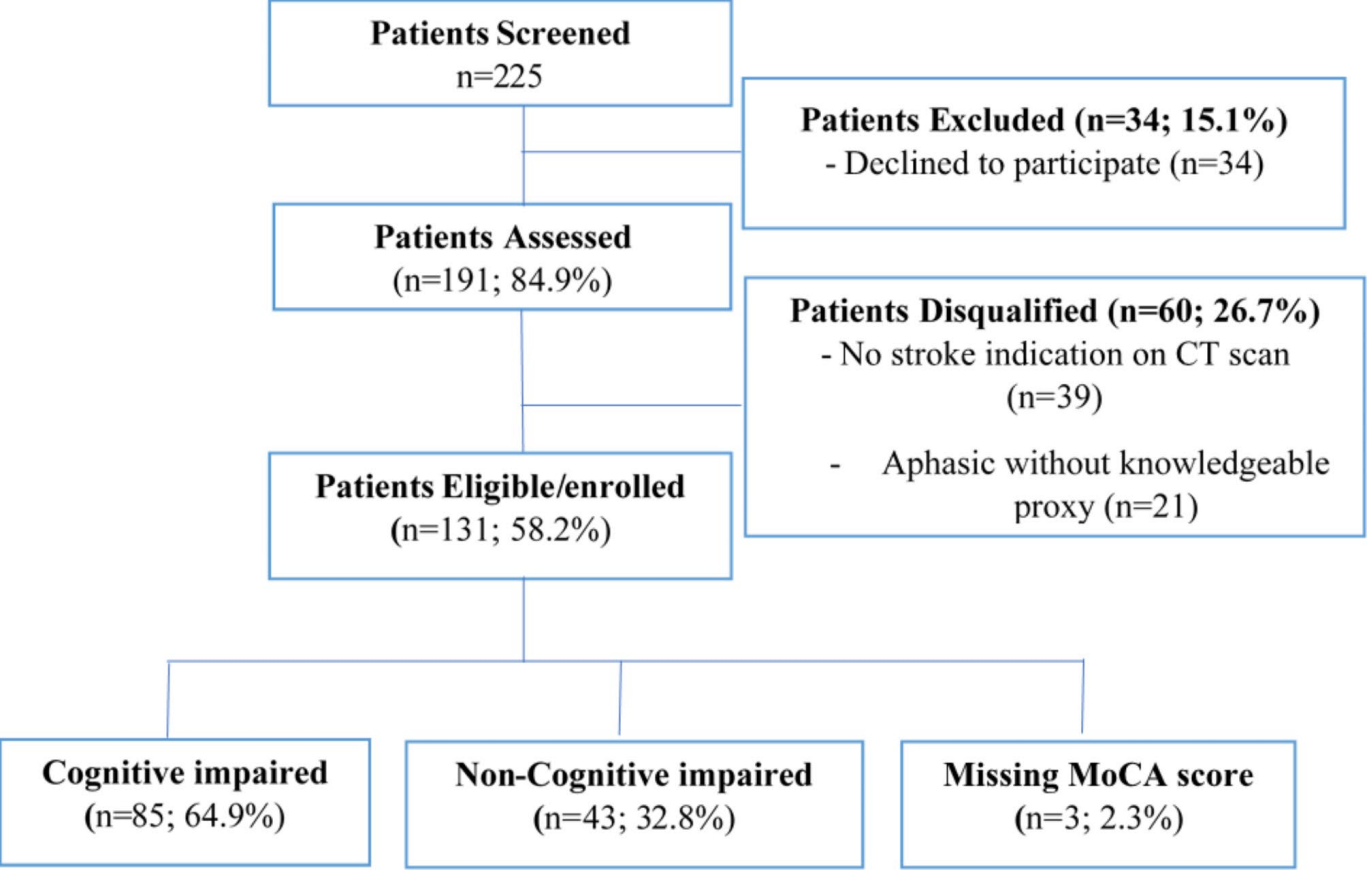
Consort Flow Diagram

131 stroke patients were enrolled (age range 20 to 91-years) including 72 (54.2%) women. Of these, 3 did not have a MOCA score and were excluded from further cognition analyses. Figure 1 gives the consort flow diagram.

The majority (71.8%) had an ischemic stroke and 28.1% had a hemorrhagic stroke. Using the TOAST classification, 42.5%, had large vessel atherosclerosis. No patient had stroke of undetermined etiology. A total of 46.6% were retired or not working and 47.3% reported either living alone or with distant relatives. The average age of participants was 57.8 ± 14.7 years with 56.5% younger than 60-years; 50.4% reported a minimum of a high school education or higher. From patient medical records, hypertension and atrial fibrillation were present in 69.4% and 13% of the study population, respectively. Table 1 gives the demographic and clinical characteristics of the study population.

**Table 1:** Study Group Characteristics

Characteristic	Frequency (%)
Overall	131 (100%)
Age (Mean (SD), [Range]) y	57.8(14.7); [20–91]
*Age group ≤ 60y*	74 (56.5%)
*Age group > 60y*	57 (43.5%)
***Sex***: *Female*	72 (54.9%)
***Sex***: *Male*	59 (45.0%)
*High Education*	66 (50.4%)
*Low Education*	65 (49.6%)
*Marital status: Married*	79 (60.3%)
*Marital status: Single/Divorced/Widowed*	52 (39.7%)
*Work status: Working*	61 (46.6%)
*Work status: Not Working*	70 (53.4%)
*Living status: Alone/with relatives*	62 (47.3%)
*Living status: With Partner/Spouse*	69 (52.7%)
**Assessments**	
*Cognitive Impairment*: *No*	43 (33.6%)
*Cognitive Impairment*: *Yes*	85 (66.4%)
*Stroke type: Ischemic*	94 (71.8%)
*Stroke type: Hemorrhagic*	37 (28.2%)
**TOAST Ischemic Stroke Subtypes**	
*Large vessel atherosclerosis*	40 (42.6%)
*Cardioembolic*	18 (19.1%)
*Small vessel occlusion*	25 (26.6%)
*Stroke of undetermined cause*	11 (11.7%)
**Lipid Profile**	**Mean (SD), [Range]**
*HDL*	1.5(0.8); [0.5–4.8]
*LDL*	2.9(1.1); [0.8–5.7]
*CHOL*	4.0(1.3): [1.6–7.4]
*TG*	2.1(0.9); [1.0–4.8]
**Functional Assessments**	**Mean, Mode, Median, [Range]**
*MoCA Score*	11.7, 0.0, 13.0; [0.0–28.0]
*NIHSS Score*	11.0, 10.0, 10.0; [0.0–30.0]
*MRS score*	3.3, 4.0, 4.0; [0.0–5.0]

### Frequency, patient profiles, and patterns of cognitive performance

**Fig. 2:**
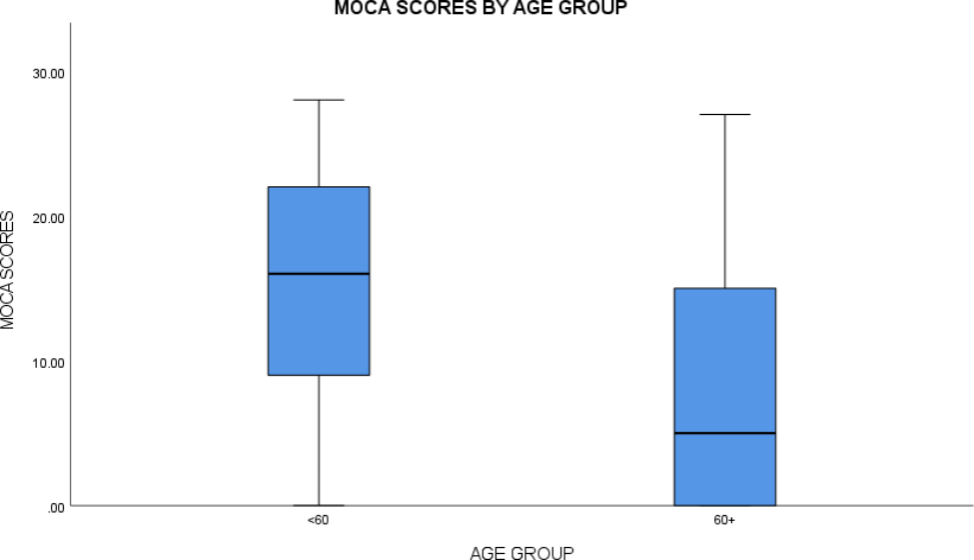
MOCA scores by Age group

The MoCA was completed by 128 (97.7%) participants. MoCA scores ranged between 0.0 and 28.0 points with a mean score of 11.7 ± 9.6-points and mode of 0.0-points. Cognitive impairment was present in 85 (66.4%), with 43 (33.6%) classified as having no cognitive impairment. Figure 2 shows MoCA score distribution for age groups < 60 and 60 + years.

70.0% of the female participants were cognitively impaired compared to 62.1% of male participants. In addition, 69.4% of the illiterate or poorly educated population were women compared to 30.6% of men (**OR** ***3.27, 95% CI 1.57–6.8; p = 0.001***).

Several variables were associated with cognitive impairment in univariate analyses including age *>* 60*yrs* (***p < 0.0001***), high LDL-C (***p = 0.048***), lower education (***p < 0.0001***), not working or retired status (***p = 0.002***), living alone or with relatives compared with spouse/partner (***p = 0.005***), higher mRs score (***p = 0.001***), Barthel scores < 12 points (***p < 0.0001***), and recurrent stroke (***p = 0.014***). Table 2 gives the results of the univariate analyses with cognitive impairment as the dependent variable.

**Table 2:** Results of Univariate Analysis, Cognitive Impairment, Crude RR by characteristics

Prevalence of Cognitive Impairment, Crude RR by characteristics			
Clinical Characteristics	No. of Cognitive impaired (%)	Crude OR, 95% CI	p-value
**Overall**	85/128 (66.4%)		
***Sex***: *Female*	49/70(70.0%)	1	
***Sex***: *Male*	36/58(62.1%)	0.70(0.34,1.47)	0.344
***Age***: *<60 years*	39/73(53.4%)	***1***	
***Age***: *60 + years*	46/55(83.6%)	***4.46(1.91,10.42)***	***< 0.0001***
*High Education*	34/66(51.5%)	***1.00***	
*Low Education*	51/62(82.3%)	***4.36(1.94,9.82)***	***< 0.0001***
*Status: Married*	47/78(60.3%)	1.00	
*Status: Single/Divorced/Widowed*	38/50(76.0%)	2.09(0.95,4.61)	0.066
*Work status: Working*	37/68(54.4%)	***1.00***	
*Work status: Not Working*	48/60(80.0%)	***3.36(1.52,7.41)***	***0.002***
*Living status: Alone/with relatives*	48/61(78.7%)	***1.00***	
*Living status: With Partner/Spouse*	37/67(55.2%)	***0.33(0.15, 0.73)***	***0.005***
*NIH score: Minor to no stroke (0–4)*	31/45(68.9%)	1.00	
*NIH score: Moderate to severe stroke (5–20)*	54/83(65.1%)	0.84(0.39,1.83)	0.661
*MRS score: Good Outcome (0–2)*	14/34(41.2%)	***1.00***	
*MRS score: Poor Outcome (3–5)*	62/83(74.7%)	***4.22(1.82,9.80)***	***0.001***
*Barthel score: Mild to no dependence (12–20)*	29/59(49.2%)	***1.00***	
*Barthel score: Severe to total dependence (< 12)*	56/69(81.2%)	***4.46(2.02,9.80)***	***< 0.0001***
*Stroke type: Hemorrhagic*	22/37(59.5%)	1.00	
*Stroke type: Ischemic*	63/91(69.2%)	1.53(0.69,3.39)	0.289
*High BP: No*	50/70(71.4%)	1.00	
*High BP: Yes*	35/58(60.3%)	0.61(0.29,1.27)	0.186
*Recurrent stroke: No*	61/100(61.0%)	***1.00***	
*Recurrent stroke: Yes*	24/28(85.7%)	***3.84(1.24,11.90)***	***0.014***
*Atrial Fibrillation: No*	70/110(63.6%)	1.00	
*Atrial Fibrillation: Yes*	15/18(83.3%)	2.86(0.78,10.47)	0.101
*Cholesterol: Low*	63/97(64.9%)	1.00	
*Cholesterol: High*	22/31(71.0%)	1.32(0.55,3.18)	0.537
*LDL: Low*	30/53(56.6%)	***1.00***	
*LDL: High*	55/75(73.3%)	***2.11(1.00,4.44)***	***0.048***

In a multivariate analysis to identify variables independently associated with cognitive impairment adjusted for age, sex and stroke severity, low education (***p = 0.016***), high mRS scores (***p < 0.001***) and high LDL-C (***p = 0.024***) were associated with moderate to severe cognitive impairment (Table 3). Associations with marital status, work status, living status, type of stroke, hypertension, and atrial fibrillation were not significant.

**Table 3:** Results of Multi-variate Analysis, Cognitive Impairment, Adjusted Crude RR by characteristics

Prevalence of Cognitive Impairment, Adjusted Crude RR by characteristics			
Clinical Characteristics	No. of Cognitive impaired (%)	Adjusted* Crude OR, 95% CI	*p-value
**Overall**	85/131 (64.9%)		
**Education**			
*High*	34/66(51.5%)	***1***	
*Low*	51/62(82.3%)	***3.23(1.25,8.33)***	***0.016***
**Marital Status**			
*Married*	47/78(60.3%)	1	
*Single/Divorced/Widowed*	38/50(76.0%)	1.69(0.65,4.35)	0.285
**Work Status**			
*Working*	37/68(54.4%)	1	
*Not Working*	48/60(80.0%)	1.86(0.68,5.08)	0.228
**Living Status**			
*Alone/with relatives*	48/61(78.7%)	1	
*With Partner/Spouse*	37/67(55.2%)	0.46(0.19,1.15)	0.096
**MRS score**			
*Good Outcome (0–2)*	14/34(41.2%)	***1***	
*Poor Outcome (3–5)*	62/83(74.7%)	***1.84(1.28,2.63)***	***< 0.001***
**Barthel Score**			
*Mild to no dependence (12–20)*	29/59(49.2%)	1	
*Severe to total dependence (< 12)*	56/69(81.2%)	2.68(0.94,7.70)	0.067
**Stroke Type**			
*Hemorrhagic*	22/37(59.5%)	1	
*Ischemic*	63/91(69.2%)	1.03(0.41,2.56)	0.953
**High BP**			
*No*	50/70(71.4%)	1	
*Yes*	35/58(60.3%)	0.69(0.30,1.61)	0.397
**Recurrent stroke**			
*No*	61/100(61.0%)	1	
*Yes*	24/28(85.7%)	3.57(0.91,14.29)	0.068
**Atrial Fibrillation**			
*No*	70/110(63.6%)	1	
*Yes*	15/18(83.3%)	1.09(0.24,4.98)	0.911
**Cholesterol**			
*Low*	63/97(64.9%)	1	
*High*	22/31(71.0%)	2.47(0.86,7.07)	0.093
**LDL**			
*Low*	30/53(56.6%)	***1***	
*High*	55/75(73.3%)	***2.74(1.14,6.56)***	***0.024***

*Adjusted for age, sex and stroke severity

## Discussion

We found a high frequency of post-stroke cognitive impairment assessed a minimum of 3-months after stroke in a consecutive series of patients evaluated at the primary referral hospital in Uganda. The overall frequency of 66.4% is higher than a previous 43% estimate in this population [31]. This previous study, conducted in 2005, involved a smaller sample of participants (n = 77), and the disparity may be due to differences in study populations. Our result is consistent with reports from Nigeria (67.4%), Ghana (72.8%), Korea (62.6%), and Norway (52.1%) [3, 32, 33].

In multivariable analyses, we included variables significant in the univariate analyses and adjusted for potential confounders identified in other studies (i.e., age, sex, and stroke severity) [24]. Our univariate analysis found that age > 60-years was associated with post stroke cognitive impairment, consistent with prior studies in other populations [22]. In contrast to some other studies [34, 35], we found no associations between MCI and the independent variables of sex and stroke severity (as measured by NIHSS scores). The lack of associations may be related to the timing of our assessments, the type of cognitive assessments, and differences in educational attainment between study populations. The extent of formal education reduces cognitive impairment in the setting f vascular dementia, Alzheimer’s disease, and mild cognitive impairment [3, 36]. A higher educational level increases the brain’s cognition reserve, which may lead to improved compensation with aging and brain injury [22].

We found that higher levels of LDL-C were independently associated with greater cognitive impairment. This is consistent with a cross-sectional analysis from four U.S. cities which found an association between increasing LDL-C levels and greater cognitive impairment [10, 37]. Additionally, findings from the Northern Manhattan Stroke Study showed that higher LDL-C was associated with the risk of incident vascular dementia [10, 38]. The mechanisms underlying the association between LDL-C and cognitive impairment are unknown, and different explanations have been proposed [8, 39, 40]. A recent study showed that LDL -C activates the secretion of pro- inflammatory mediators such as tumor necrosis factor alpha and interleukin-6 and decreased the BBB membrane injury, a contributory factor to the development and progression of cognitive impairment [41, 42]. Alternatively, high LDL-C may be associated with more extensive, including silent ischemic brain injury that could affect cognition [43, 44].

In contrast to observational studies indicating that hypertension is correlated with the prevalence of dementia, we found no relationship between high blood pressure and cognitive impairment [45]. Because all of our participants had a stroke, which is strongly associated with hypertension, the relationship between hypertension and cognition may have been attenuated. Atrial fibrillation was not associated with cognitive impairment in our study. The reported relationship between atrial fibrillation and cognitive impairment may have similarly been reduced because of the relationship between it and ischemic brain injury as well as atrial fibrillation and brain magnetic resonance imaging abnormalities [46]. This could also be explained by the small number of participants who reported a history of atrial fibrillation (13%).

High mRS score (i.e., 3 − 5) was independently associated with cognitive impairment. Scores 3–5 are associated with high to total patient handicap and dependence on others in performing activities of daily living [47]. Stroke survivors who are physically dependent and more impaired tend to perform poorly on cognitive tasks, possibly due to having larger areas of brain injury [48]. We found no overall association between stroke subtype and cognitive impairment. Cognition may be differentially affected depending on both the extent of brain injury and affected brain structures, data that were not reflected in our analyses.

Functional dependence as assessed by the BI predicted cognitive impairment as a single variable; however, the association between BI and cognitive impairment was not significant when controlling for potential confounders in the multivariate analysis. This could be due to collinearity between the independent variables or it could also be a result of the limitations of the BI. Functional dependence may be affected by other environmental, social, and individual adaptations that contribute to activities of daily living [49]. In addition, the sensitivity of the BI to distinguish between high and low performers is limited [50]. Only severe cognitive impairment may interfere with tasks reflected in the BI.

Our study has several limitations. [1] Our relatively small sample size could reduce our statistical power to identify associations of lower effect size. Because of the cross-sectional design, we cannot assess causal relationships between putative predictors and post-stroke cognitive impairment. [2] Dementia involves a change in function and cognition, but we did not conduct longitudinal assessments and in some cases, relied on proxy reports for some variables [3]. There is also a potential inception cohort bias because our sample included only participants seen at a single national referral hospital. [4] The MoCA is not validated in a sub-Saharan African population, and therefore does not take account of certain linguistic and cultural variables which may affect interpretation of the MoCA across different countries. In addition, participants’ educational backgrounds affect the results of cognitive assessments, including the MoCA, resulting in a possible overestimation of cognitive impairment among those who have lower levels of formal education. Using a MoCA cut-off of 19 to classify cognitive impairment in this population rather than the more common 26 points, however, mitigates this effect. [5] Most of the study participants had a relatively mild stroke and our results may not be generalizable to those with a more severe or very mild stroke, or to those who have specific deficits (e.g., aphasia) that precluded cognitive assessment. [6] Brain MRI scans were not available, and we therefore could not assess the potential impact of silent lacunar infarctions or white matter injury, which could affect poststroke cognition [51].

Limitations notwithstanding, our study provides unique data on the frequency and risk factors for post-stroke cognitive impairments in Uganda. The data are helpful for creating public awareness, influencing policy recommendations, and guiding further research that may lead to effective interventions.

## Conclusion

We found a high prevalence of cognitive impairment (64.9%) among Ugandan patients who had a stroke. Increasing age, low level of education, functional handicap, and high LDL cholesterol were independently associated with cognitive impairment. Our findings underscore the high burden and need for awareness of cognitive impairment in post stroke populations in the sub-Saharan region and serve to emphasize the importance of cognitive assessments as part of routine clinical evaluation of patients who have had a stroke.

## Data Availability

The datasets used and/or analysed during the current study are available from the corresponding author on reasonable request.
